# Differential Transcriptome Responses to Aflatoxin B_1_ in the Cecal Tonsil of Susceptible and Resistant Turkeys

**DOI:** 10.3390/toxins11010055

**Published:** 2019-01-18

**Authors:** Kent M. Reed, Kristelle M. Mendoza, Roger A. Coulombe

**Affiliations:** 1 Department of Veterinary and Biomedical Sciences, College of Veterinary Medicine, University of Minnesota, Saint Paul, MN 55108, USA; mendo008@umn.edu; 2 Department of Animal, Dairy and Veterinary Sciences, College of Agriculture and Applied Sciences, Utah State University, Logan, UT 84322, USA; roger@usu.edu

**Keywords:** Poultry, Turkey, Transcriptome, Aflatoxin B_1_, Cecal Tonsil, Cecum, RNAseq

## Abstract

The nearly-ubiquitous food and feed-borne mycotoxin aflatoxin B_1_ (AFB_1_) is carcinogenic and mutagenic, posing a food safety threat to humans and animals. One of the most susceptible animal species known and thus a good model for characterizing toxicological pathways, is the domesticated turkey (DT), a condition likely due, at least in part, to deficient hepatic AFB_1_-detoxifying alpha-class glutathione S-transferases (GSTAs). Conversely, wild turkeys (Eastern wild, EW) are relatively resistant to the hepatotoxic, hepatocarcinogenic and immunosuppressive effects of AFB_1_ owing to functional gene expression and presence of functional hepatic GSTAs. This study was designed to compare the responses in gene expression in the gastrointestinal tract between DT (susceptible phenotype) and EW (resistant phenotype) following dietary AFB_1_ challenge (320 ppb for 14 days); specifically in cecal tonsil which functions in both nutrient absorption and gut immunity. RNAseq and gene expression analysis revealed significant differential gene expression in AFB_1_-treated animals compared to control-fed domestic and wild birds and in within-treatment comparisons between bird types. Significantly upregulated expression of the primary hepatic AFB_1_-activating P450 (*CYP1A5*) as well as transcriptional changes in tight junction proteins were observed in AFB_1_-treated birds. Numerous pro-inflammatory cytokines, *TGF-β* and *EGF* were significantly down regulated by AFB_1_ treatment in DT birds and pathway analysis suggested suppression of enteroendocrine cells. Conversely, AFB_1_ treatment modified significantly fewer unique genes in EW birds; among these were genes involved in lipid synthesis and metabolism and immune response. This is the first investigation of the effects of AFB_1_ on the turkey gastro-intestinal tract. Results suggest that in addition to the hepatic transcriptome, animal resistance to this mycotoxin occurs in organ systems outside the liver, specifically as a refractory gastrointestinal tract.

## 1. Introduction

Aflatoxin B_1_ (AFB_1_) is a hepatotoxic, hepatocarcinogenic and immunosuppressive mycotoxin commonly found in food and feed, especially corn [1]. Poultry are particularly sensitive to the toxic effects of AFB_1_ and commercial domesticated turkeys are perhaps the most susceptible animal thus far studied [2,3]. Exposure to AFB_1_ through contaminated feed is practically unavoidable and can result in reduced feed intake, weight gain and feed efficiency and increased mortality, hepatotoxicity and GI hemorrhaging (reviewed in Monson et al. [4]). As a potent immunotoxin, AFB_1_ suppresses cell-mediated, humoral and phagocytic immunological functions, thereby increasing susceptibility to bacterial and viral diseases [5,6,7].

In contrast to their modern domesticated counterparts, wild turkeys are relatively resistant to aflatoxicosis [8]. Metabolism of AFB_1_ requires bioactivation by hepatic cytochrome P450s (CYPs) to the electrophilic exo-AFB1-8,9-epoxide (AFBO), which is catalyzed primarily, at pharmacological concentrations by the high-efficiency CYP1A5 and to a minor extent by the lower-affinity CYP3A37 which predominates only at high, environmentally-irrelevant substrate concentrations [9]. In most animals, AFBO is detoxified primarily by hepatic glutathione S-transferases (GSTs) [3]. The most likely mechanism for the extreme susceptibility in domesticated turkeys is dysfunctional hepatic GSTs rendering them unable to detoxify AFB_1_ [10,11,12,13,14]. In this regard, domesticated turkeys closely resemble humans in that they also lack hepatic alpha-class GSTs (GSTA) with high activity toward AFB_1_ (seen in mice and rats) suggesting that turkeys may represent a better model to study aflatoxin toxicology than either of these rodent species [9]. Expression of GSTA in the intestine and the potential for extra-hepatic bioactivation and metabolism of AFB_1_ in turkeys is unknown.

To better understand the response of the domestic turkey to AFB_1_ exposure, we initiated transcriptomic analysis of AFB_1_-challenged domestic birds [15], where genes and gene pathways in the liver were significantly dysregulated by dietary AFB_1_ challenge, such as pathways associated with cancer, apoptosis, cell cycle and lipid regulation. These changes reflect the molecular mechanisms underlying DNA alkylation and mutation, inflammation, proliferation and liver damage in aflatoxicosis. Analysis of spleen tissues from the same birds examined in the Monson et al. [15] study found that short AFB_1_ exposure suppressed innate immune transcripts, especially from antimicrobial genes associated with either increased cytotoxic potential or activation-induced cell death during aflatoxicosis [16].

The differential response of domestic and wild turkey to AFB_1_ was examined in a controlled feeding trial [17]. Analysis by RNAseq of the hepatic transcriptome found genes dysregulated as a response to toxic insult with significant differences observed between these genetically distinct birds in the expression of Phase I and Phase II drug metabolism genes. Genes important in cellular regulation, modulation of apoptosis and inflammatory responses were also affected. Unique responses in wild birds were seen for genes that negatively regulate cellular processes, serve as components of the extracellular matrix or modulate coagulation factors. Wild turkey embryos also showed differential AFB_1_ effects compared to their commercial counterparts presumably due to lower levels of AFBO [18]. When treated with AFB_1_, embryos showed up-regulation in cell cycle regulators, Nrf2-mediated response genes and coagulation factors [18]. Results of these studies supported the hypothesis that the reduced susceptibility of wild turkeys is related to higher constitutive expression of *GSTA3*, coupled with an inherited (genetic) difference in functional gene expression in domesticated birds.

The molecular basis for the differences in AFB_1_ detoxification observed between domesticated commercial and wild birds has been extensively studied in our laboratories. However, extra-hepatic effects, such as those occurring at the site of initial toxicant exposure, the intestine, are needed to fully understand the systemic effects of AFB_1_ in this susceptible species. Unlike many mycotoxins, AFB_1_ is efficiently absorbed (>80%) in the avian upper gastrointestinal tract (GIT) [19]. Recent studies of broiler chickens have found conflicting evidence for the potential impact of AFB_1_ on gut permeability, from no effect [20] to increased permeability [21]. The avian small intestine is a primary site of nutrient absorption [22] but is often overlooked from an immunological perspective. The cecal tonsils are the largest aggregates of avian gut-associated lymphoid tissue, yet basic information on gene expression in the cecal tonsil is lacking in the turkey. This study focused on the effects of dietary AFB_1_ on gene expression in the turkey GIT and specifically the region at the junction of the distal ileum and cecum (the cecal tonsil region) that functions in AFB_1_ absorption and gut immunity. The purpose of this study was to examine the transcriptomic response of the cecal tonsil region of the turkey intestine to dietary AFB_1_ treatment and contrast these in susceptible (domesticated) and resistant (wild) birds. 

## 2. Results

**Figure 1:**
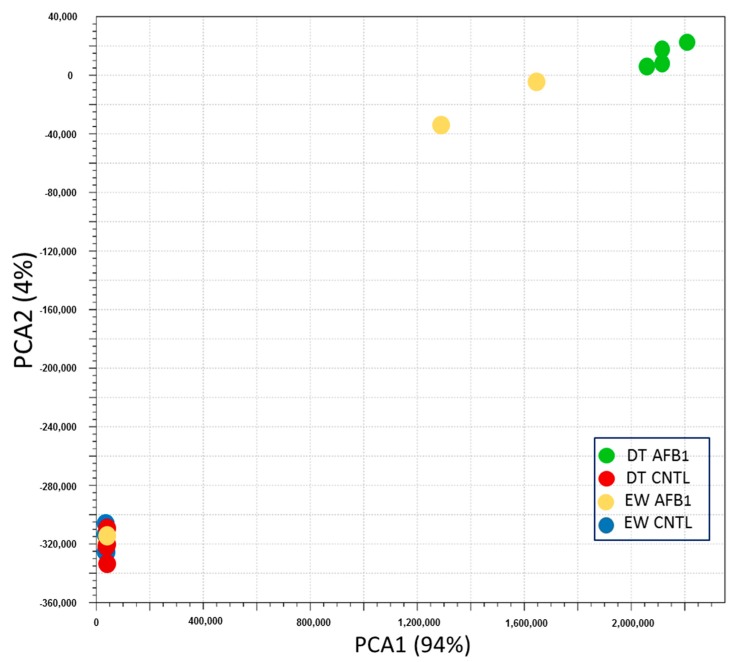
Principal component analysis (PCA) of by-total normalized RNAseq read counts. For each treatment group, sample to sample distances (within- and between-treatments) are illustrated on the first two principle components.

The effects of AFB_1_ on body weight and liver mass are summarized in a companion study of hepatic gene expression [17]. Sequencing produced from 9.8M to 14.2M reads per library (average 12.7 million) (Table S1). Data are deposited in the NCBI’s Gene Expression Omnibus (GEO) repository as SRA BioProject 346253. Median Q scores of the trimmed and filtered reads ranged from 36.5 to 37.7 among the forward and reverse reads. The number of reads per treatment group ranged from 10.9 to 12.8M with the mean number for EW birds being slightly higher than for the DT birds (12.6M verses 11.2M). Over 90% of the quality-trimmed reads mapped to the annotated turkey gene set (NCBI Annotation 101) and the vast majority of reads (average 85.2%) mapped concordantly (Table S1). Based on mapping, the estimated mean insert size of the libraries was 195.4 ± 15.8 bp. Variation in mapped reads among the treatment groups was visualized by PCA (Figure 1). Samples (AFB_1_ treatment/CNTL) generally clustered distinctly by treatment group within the space defined by the first two principal components. The exceptions were two EW AFB_1_ samples (EW1C and EW3C) that clustered with the control birds. The relationships among groups was reiterated in the hierarchical clustering of groups by Euclidean distance and heat map of co-expressed genes (Figure S1). This indicates the main effect underlying this study is AFB_1_ treatment.

**Figure 2:**
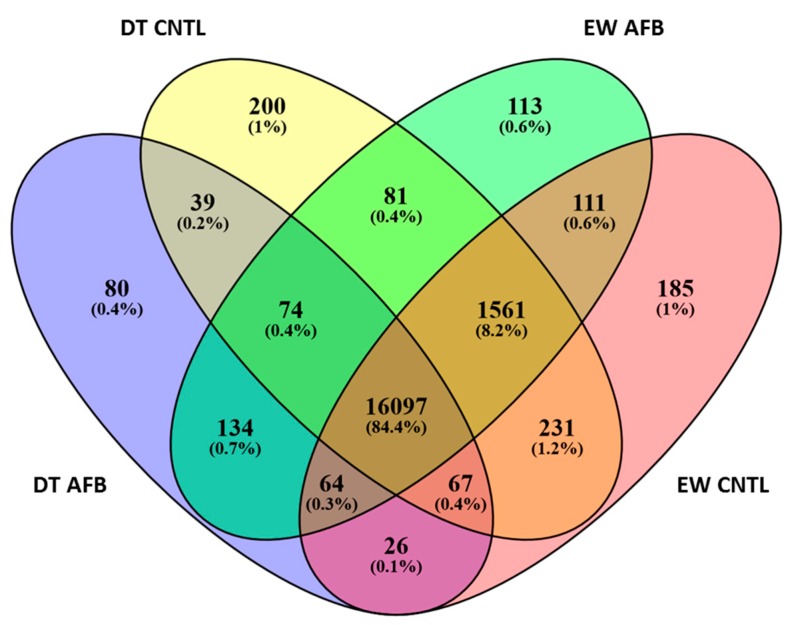
Distribution of expressed genes in turkey cecal tonsil by treatment group.

Evidence of expression (mapped reads ≥ 1.0 in at least one individual) was detected for 19,754 genes (tRNAs excluded) with an average of 17,261 genes observed per individual (Tables S1 and S2). When qualified (by-total normalized read count ≥ 3.0), the number of expressed genes averaged 16,132 per individual (76.79% of the turkey gene set) with an average of 17,877 expressed genes per treatment group. The numbers of observed and expressed genes were higher for control groups than for AFB_1_-treatment groups of both EW and DT. A total of 16,097 genes (84.4%) was co-expressed among all groups and the number of co-expressed genes within the EW and DT lines was 17,833 and 16,277, respectively (Figure 2). Each treatment group had a distinct set of uniquely expressed genes, with the numbers being greater for the control groups (200 and 185) compared to the AFB_1_ groups (80 and 113) (Figure 2).

### 2.1. Differential Gene Expression

#### 2.1.1. AFB_1_ Treatment Effects 

**Figure 3:**
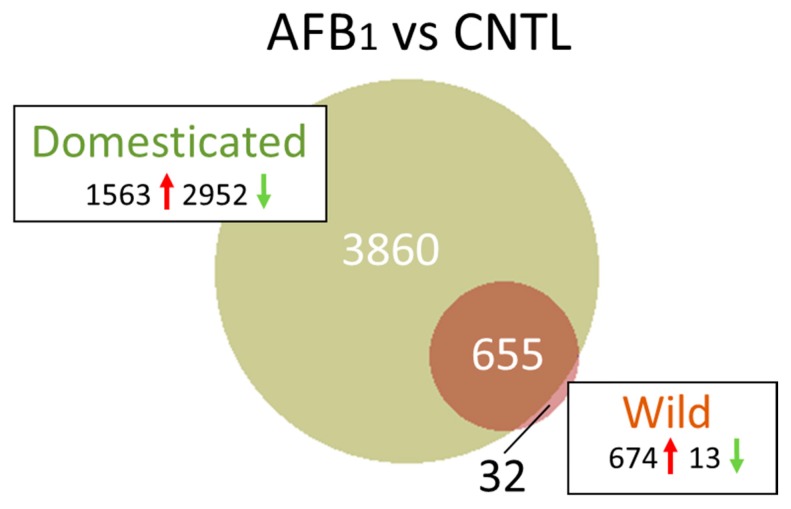
Distribution of differentially expressed genes in the turkey. For each comparison, the number of significant genes (FDR *p*-value < 0.05 and |log_2_FC| > 2.0) shared or unique to each treatment are indicated in the Venn diagram. Circle size is proportional to the number of genes and direction of expression change (↑ or ↓) is given for each group.

The full list of genes showing significant differential expression (DE) in pairwise treatment comparisons is provided in Table S3. In comparison of DT birds exposed to AFB_1_ (DTAFB) with control-fed birds (CNTL) DE was observed for 11,237 genes in the cecal tonsil (FDR *p*-value < 0.05). Of these, 7568 had |log_2_FC| > 1.0 and 4515 had |log_2_FC| > 2.0 (Table 1). The number of DE genes was considerably fewer for the AFB_1_-treated EW turkeys (703 with FDR *p*-value < 0.05 and 687 genes with |log_2_FC| > 2.0). In DT birds, the majority (65.4%) of DEGs were down regulated (Figure 3) although 48 of the 50 genes with the greatest fold change were up regulated (Table S4). In contrast, 98% of the DEGs in AFB_1_-treated EW birds were up regulated. Combined, 655 DEGs were shared in comparisons for both bird types, with 3860 being unique to DT birds and 32 unique to EW birds (Figure 3). Functional interpretation of many avian genes is based on sequence and syntenic similarity with human and other model organisms and therefore many functions are necessarily posited.

##### Shared Transcriptome Response

Among the 655 shared genes were the two phase I enzymes important in AFB_1_ metabolism (Table S3). The first, *CYP1A5* (cytochrome P450, family 1, subfamily A, polypeptide 5) was highly up regulated in both EW and DT birds treated with AFB_1_ (log_2_FC = 7.66 and 9.67, respectively). Secondly, *CYP3A37* (cytochrome P450 3A37) was significantly up regulated in only the DT birds (log_2_FC = 2.73). Studies from our laboratory have identified these as the principal turkey hepatic cytochromes responsible for efficient epoxidation of AFB_1_; CYP1A5 has highest affinity toward AFBO (low Km, high Vmax/Kcat) and bioactivates > 99% of AFB_1_ in turkey liver. In turkey, CYP3A37 (high Km, low Vm, Kcat) is only active at high environmentally-irrelevant substrate (i.e., AFB_1_) concentrations [9]. Although potential biochemical activity of GSTAs in the intestine (cecal tonsil) of turkeys is unknown, expression of *GSTA4* was significantly up regulated in both the EW and DT birds with AFB_1_ exposure (log_2_FC = 4.53 and 5.89, respectively).

DE was also observed for several members of the claudin protein family. Claudins are integral components forming the backbone of the tight junctions of epithelial and endothelial cells [23]. In EW birds, *CLDN1* (claudin 1) was up regulated by AFB_1_ (log_2_FC = 4.55), whereas *CLDN18* was down regulated (log_2_FC = −6.57) (Table S3). In DT birds, *CLDN1*, *CLDN2* and *CLDN11* were up regulated (log_2_FC = 6.04, 4.01 and 2.17, respectively) and *CLDN3*, *CLDN10*, *CLDN19* and *CLDN23* were down regulated (log_2_FC = −2.52, −7.17, −4.11, −8.05, respectively). Expression of other key tight-junction proteins, tricellulin (MARVEL domain-containing protein 2, *LOC104915344*) and occludin (*LOC104915505*), were also significantly altered in DT but with smaller fold changes (Table S3). Upregulation of membrane tight-junction proteins such as claudins, is indicative of an epithelial response in the gut lumen to AFB_1_ and may suggest that AFB_1_ could alter gut permeability and perhaps stimulate a protective response in the gut to diminish mucosal inflammation/immune defense and repair processes.

**Figure 4:**
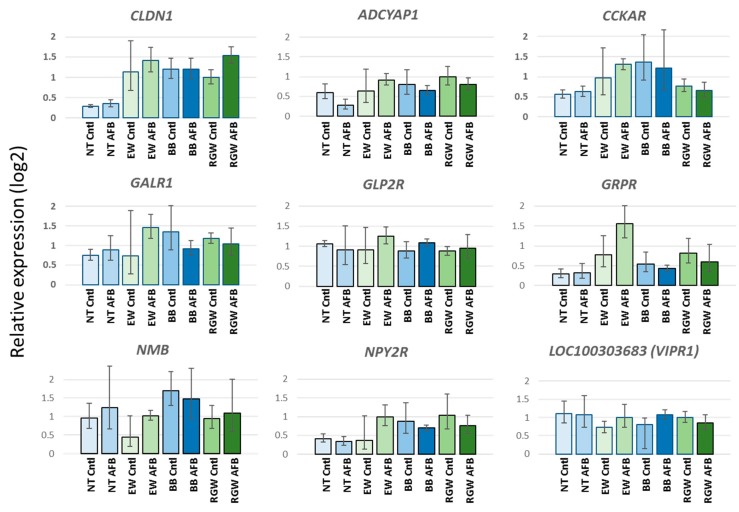
Effect of AFB_1_ on expression of genes in the IPA canonical pathway “GPCR-Mediated Integration of Enteroendocrine Signaling Exemplified by an L Cell” in the cecal tonsil of turkeys (see Figure 5).

Expression differences in *CLDN1* observed in RNAseq read counts were further tested by qRT-PCR where expression of *CLDN1* transcripts was significantly higher in EW birds compared to controls regardless of AFB_1_-treatment (Figure 4). Relative *CLDN1* expression was also similarly variable in other wild-type birds (Rio Grande Wild, RGW) where expression was comparable to that of EW birds and significantly elevated with AFB_1_ treatment. Expression in other domestic birds (broad breasted white, BB) was more similar to that of the wild birds than the Nicholas DT suggesting that the lower *CLDN1* expression observed in the Nicholas DT birds may have a genetic component.

Only two of the 655 shared DEGs (*ATP12A* and *RSAD2*) in the RNAseq data showed differences in the directionality of expression. *ATP12A* (ATPase, H^+^/K^+^ transporting, non-gastric, alpha polypeptide) was down regulated (log_2_FC = −2.83) in DT and up regulated (log_2_FC = 4.69) in EW birds. Similarly, *RSAD2* (radical S-adenosyl methionine domain containing 2) was down regulated (log_2_FC = −3.47) in DT and up regulated (log_2_FC = 3.23) in EW. Two additional loci (*SCD*, stearoyl-CoA desaturase [delta-9-desaturase]) and a ncRNA (*LOC104914677*) had a similar directional expression pattern, with significant up regulation in EW with AFB_1_ treatment and down regulation in DT, however the log_2_FC in the DT birds was below 2.0. ATP12A is a member of the P-type cation transport ATPase family and in humans is involved in tissue-specific potassium absorption [24]. RSAD2 is an interferon inducible antiviral protein and has been shown in human cell lines to inhibit secretion of soluble proteins [25]. In mammals, SCD has a regulatory role in the expression of genes involved in lipogenesis and is important in mitochondrial fatty acid oxidation and energy homeostasis [26].

Nine of the 655 DEGs were significantly down regulated in both DT and EW with AFB_1_ treatment. These included *GGT1* (gamma-glutamyltransferase 1), *OTOR* (otoraplin), *PLIN1* (perilipin 1), *RSPH14* (radial spoke head 14 homolog), *SLC34A2* (solute carrier family 34, member 2), *LOC100550279* (fatty acid-binding protein, adipocyte-like [*FABP4*-like]), *LOC104909385* (erythroblast NAD(P)(+)--arginine ADP-ribosyltransferase pseudogene), *LOC104913555* (gamma-glutamyltranspeptidase 1-like) and *TNFRSF13C* (tumor necrosis factor receptor superfamily, member 13C). Genes of particular interest in the GI tract include Perilipin 1 and fatty acid-binding protein (*LOC100550279*) that are involved in lipid transport and metabolism in human adipocytes [27]. SLC34A2 is a sodium-dependent phosphate transporter with an inverse pH dependence [28]. It is expressed in several mammalian tissues of epithelial origin including lung and small intestine and may be the main phosphate transporter in the brush border membrane. The B-cell activating factor TNFRSF13C is known to promote survival of mammalian B-cells in vitro and is a regulator of the peripheral B-cell population [29].

Functional gene classification of the 655 shared DEGs with DAVID identified 10 enriched gene clusters (Table S5). The cluster with the highest enrichment score included members of the serpin family of protease inhibitors (*SERPINA10*, *SERPINC1*, *SERPIND1*, *SERPINF2* and *SERPING1*) that control many inflammation and coagulation processes. Other enriched clusters included complement components, mannan-binding lectin serine peptidase 1 and 2 (*MASP1*, *MASP2*), the (C4/C2 activating components) and coagulation factors F2, F7, F9 and F10. PANTHER overrepresentation tests found greatest fold enrichment for biological processes indicative of the dual absorption/immunity roles of the small intestine. Complement activation (GO:0001867) and regulation of intestinal absorption (GO:1904729, 1904478, 0030300) were significantly enriched as was cholesterol homeostasis GO:0042632) as exemplified by up regulation of several genes (*ABCG5*, *ABCG8*, *ANGPTL3*, *APOA1*, *APOA4*, *APOA5*, *CETP*, *EPHX2*, *G6PC*, *LIPC* and *LPL*).

##### Unique Transcriptome Responses

Domesticated birds showed the greatest AFB_1_ gene response with 3860 unique DEGs (Figure 3). Genes showing the highest differential response (Table S4) were enriched for those encoding proteins with signal peptides and Serpins. DEGs with the greatest up regulation included *INHBC* (inhibin, beta C, log_2_FC = 13.63), claudin-19-like (*LOC100544298*, log_2_FC = 12.56), *TTC36* (tetratricopeptide repeat domain 36, log_2_FC = 12.28) and three ncRNAs (*LOC104913410*, *LOC104915491*, *LOC10491649*, log_2_FC =12.74 to 13.15), *SMIM24* (small integral membrane protein 24, log_2_FC = −12.48) and *SLC10A2* (solute carrier family 10 [sodium/bile acid cotransporter], member 2, log_2_FC = −12.07). Expression of *GSTA3* was significantly lower in DT birds treated with AFB_1_ compared to controls (log_2_FC = −2.33). Other αGSTs (*GSTA1* and *GSTA2*) were significantly up regulated but with lower fold change (log_2_FC < 2.0, Table S3).

**Figure 5:**
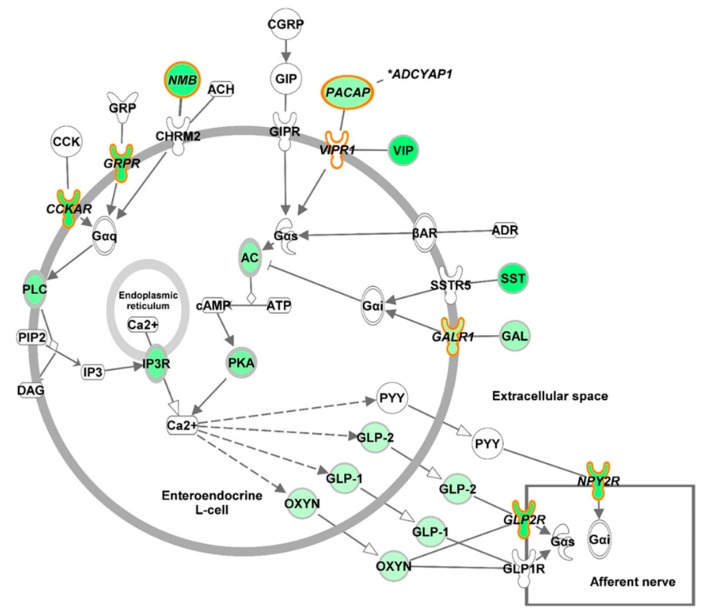
Differential expression of genes in the IPA canonical pathway “GPCR-Mediated Integration of Enteroendocrine Signaling Exemplified by an L Cell.” Genes with significantly lower expression in domesticated turkeys relative to Eastern wild birds after AFB_1_ treatment are denoted in green. Genes tested by qRT-PCR are outlined in orange (Figure 4).

Over 650 of the 3860 DEGs were functionally clustered (DAVID enrichment score 24.96) as having membrane or transmembrane UniProt keywords. The majority of these (518, 77.9%) were down regulated as an effect of AFB_1_ treatment. Several alpha-1-antitrypsin-like loci were significantly up regulated consistent with a response to acute inflammation. Analysis of the 3860 unique genes in IPA found the most significant canonical pathways to be Axonal Guidance Signaling (-log(*p*-value) = 8.65), Hepatic Fibrosis / Hepatic Stellate Cell Activation (8.24), GPCR-Mediated Integration of Enteroendocrine Signaling Exemplified by an L Cell (7.33) and Calcium Signaling (7.28). DEGs in these pathways were almost exclusively down regulated in AFB_1_-treated birds. This effect is dramatically illustrated for the in the IPA canonical pathway “GPCR-Mediated Integration of Enteroendocrine Signaling Exemplified by an L Cell” (Figure 5) suggesting suppression in domesticated birds of enteroendocrine cells that produce and release gastrointestinal hormones such as glucagon-like peptides, peptide YY and oxyntomodulin that participate in nutrient sensing and appetite regulation and peptides to activate nervous responses [30].

Differential expression differences in genes of the “GPCR-Mediated Integration of Enteroendocrine Signaling Exemplified by an L Cell” pathway observed in RNAseq read counts were further tested in eight genes by qRT-PCR. These included *ADCYAP1* (adenylate cyclase activating polypeptide 1), *CCKAR* (cholecystokinin A receptor), *GALR1* (galanin receptor 1), *GLP2R* (glucagon-like peptide 2 receptor), *GRPR* (gastrin-releasing peptide receptor), *NMB* (neuromedin B), *NPT2R* (neuropeptide Y receptor Y2) and *VIPR1* (*LOC100303683*, vasoactive intestinal polypeptide receptor). With the exception of *VIPR1*, each of these genes showed lower expression in AFB_1_-treated DT birds as compared to treated EW birds. The VIPR1 receptor was selected as it is downstream of two affected genes (*ADCYAP1* [*PACAP*] and *VIP*) in the pathway. With the exception of *NMB* and *VIPR1*, expression of the selected genes in EW birds was greater than in DT (domestic Nicholas turkey) consistent with RNAseq results (Figure 4). Disparate results between qRT experiments and RNAseq may be attributed to the higher efficiency of qRT-PCR in sampling genes with low average expression such as *NMB*. In the case of *ADCYAP1*, *CCKAR* and *GRPR* expression was also greater in the untreated EW birds relative to untreated DT birds. As expected, little variation was observed in *VIPR1*. Relative expression of these genes was also tested in the other commercial-type (broad-breasted white, BB) and wild-type birds (Rio Grande subspecies, RGW). Comparable expression results were seen for *ADCYAP1* and *GRPR*. Expression of 3 genes in the BB birds (*CCKAR*, *GALR1* and *NPY2R*) was elevated as compared to the DT group with levels more similar to the EW and RGW groups (Figure 4).

Only 32 DEGs were found unique to the wild turkey in the AFB_1_ versus CNTL RNAseq comparison (Figure 3). The majority (28, 87.5%) were up regulated in the AFB_1_-treated birds. Included among these are genes involved in lipid synthesis and metabolism (exemplified by *ACSBG2*, *ANGPTL4* and *SCD*) and immune response (*IRG1* [immunoresponsive 1 homolog], *PI3* [peptidase inhibitor 3]). A single annotation cluster (GO:0016021 integral component of membrane) was identified in DAVID that included 5 genes (*CLDN18*, *FAXDC2*, *PTPRQ*, *SCD* and *SLC23A1*). Interestingly, 29 of the 32 unique genes were also DE in the liver transcriptomes obtained from the same individuals [17] but showed opposite directional change in response to AFB_1_.

### 2.2. Wild versus Domesticated Turkey

#### 2.2.1. Control Birds

**Figure 6:**
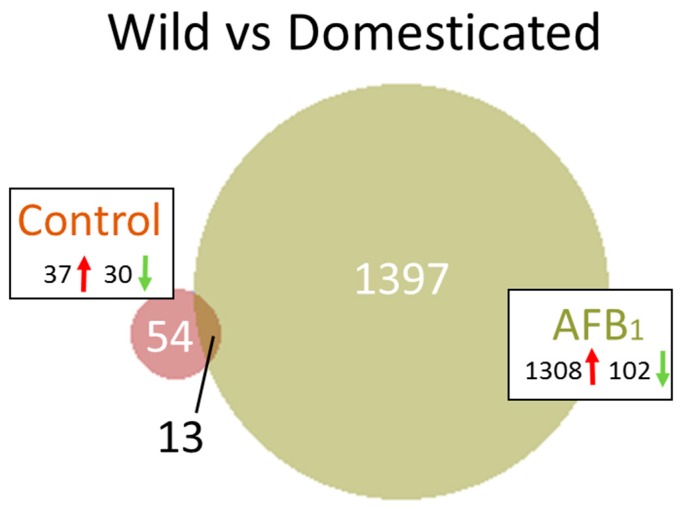
Distribution of differentially expressed genes between turkey types (wild and domesticated). For each comparison, the number of significant genes (FDR *p*-value < 0.05 and |log_2_FC| > 2.0 shared or unique to each treatment group are indicated. Circle size is proportional to the number of genes and direction of expression change (↑ or ↓) is given for each group.

Comparison of the transcriptomes of EW and DT birds in the control groups found 679 DEGs (FDR *p*-value < 0.05, log_2_FC = −7.882 to 6.715, Table 1 and Table S3), with 67 having |log_2_FC| > 2.0 (Figure 6, Table S6). Of the 67 genes, 13 were shared in common in the EW versus DT AFB_1_ comparisons (Figure 6). The shared loci included 7 genes up regulated in EW birds; (*CAMK4* [calcium/calmodulin-dependent protein kinase IV], *LOC100548321* [Pendrin], *NEFM* [neurofilament, medium polypeptide], *LOC104914065* [pendrin-like] *LINGO2* [leucine rich repeat and Ig domain containing 2], *LOC100538933* [probable ATP-dependent RNA helicase *DDX60*] and the uncharacterized *LOC100549340* [ncRNA]). This differential expression may have implications for both epithelial function and inflammatory response. For example, as an anion exchange protein, Pendrin may function to regulate active chloride transport across epithelial membranes as a chloride-formate exchanger [31]. CAMK4 is implicated in transcriptional regulation in immune and inflammatory responses [32] and DDX60 is thought to positively regulate DDX58/RIG-I- and IFIH1/MDA5-dependent type I interferon and interferon inducible gene expression [33].

Down regulated genes among the 13 shared DE loci in the EW/DT comparison included *LOC100540418* (BPI fold-containing family C protein-like [*BPIFC*]), *LOC104915630* (3 beta-hydroxysteroid dehydrogenase/Delta 5-->4-isomerase-like [*HSD3B1*]), *LOC104917314* (14-3-3 protein gamma-B) and 3 uncharacterized ncRNA loci. Two of these genes have direct implication in gut homeostasis. BPIFC is a lipid transfer/lipopolysaccharide binding protein that may help provide defense against microorganisms [34]. In humans, HSD3B1 is an important gene in the biosynthesis of hormonal steroids as it catalyzes oxidative conversion of delta-5-3-beta-hydroxysteroid precursors. Altered expression of hormones in the gut may directly influence gene expression in the gut microbiota [35].

Of the 54 DEGs unique to the control group birds slightly more (55%) were up regulated in the EW birds compared to the DT birds (Table S6). These 54 unique DEGs included integral membrane proteins (e.g., *AQP10*), cytoplasmic enzymes (*NME8*), nuclear transcriptional regulators (*HOXB5*) and secretory proteins (*GKN2*) that are typical of intestinal epithelium but without significant enrichment for any particular biological process. Greatest differential expression was seen for claudin 18 (*CLDN18*), a membrane protein that is a component of tight junction strands with higher expression in EW (log_2_FC = 6.72) than DT. Also represented were genes with immune system roles such as *DNTT* (DNA nucleotidylexotransferase), which functions in generating antigen receptor diversity and *NOS1* (nitric oxide synthase 1), a host defense effector with antimicrobial activity.

#### 2.2.2. AFB_1_ Treatment

The greatest number gene expression differences observed between the EW and DT birds occurred in the AFB_1_-treatment groups. A total of 1666 DEGs (FDR *p*-value < 0.05) were observed with 1410 having |log_2_FC| > 2.0 (Table 1). As discussed above, 13 DEGs were shared with the control comparison and 1397 were unique (Figure 6, Table S7). Interestingly, 93% of the DEGs showed higher expression in the EW birds compared to DT. Non-coding RNAs comprised 29.4% of the down regulated genes (n = 30) and 5% of the up regulated DEGs (n = 66). Greatest differential expression (up regulation) in EW compared to DT was seen for *LOC104912821* (ovostatin homolog, log_2_FC = 11.84), LOC104915655 (alpha-2-macroglobulin, *A2M*, log_2_FC = 11.4) and genes such as *SLC10A2* (solute carrier family 10 [sodium/bile acid cotransporter] member 2, log_2_FC = 11.06) and *FABP6* (fatty acid binding protein 6, log_2_FC = 10.26). Ovostatin and A2M both have endopeptidase inhibitor activity, whereas SLC10A2 and FABP6 function in bile acid metabolism. Greatest down regulation in EW compared to DT was seen for *GYG2* (Glycogenin 2, log_2_FC = −7.19) and *LOC104916581* (7-dehydrocholesterol reductase-like, log_2_FC = −5.56). In humans, GYG2 is expressed mainly in the liver and heart and is involved in initiating reactions of glycogen biosynthesis; 7-dehydrocholesterol reductase is ubiquitously expressed and helps catalyze the production of cholesterol [36,37].

Functional analysis of the 1397 unique DEGs in DAVID found highest enrichment score (14.11) for the annotation cluster “Membrane” (*p* = 4.1 × 10^−16^), which included 284 genes (Table S7). The second annotation cluster (enrichment = 5.39) contained 50 genes with immunoglobulin-like domains or Ig-like fold (homologous superfamily IPR013783, *p* = 5.7 × 10^−7^). Included were several complement proteins, interleukins and Ig superfamily members (Table S7). Additional clusters identified in DAVID included “extracellular exosome” (136 DEGs, *p* = 6.5 × 10^−3^) and “signal” (118 DEGs, *p* = 2.3 × 10^−8^). Calcium signaling was the most expressively represented Kegg pathway containing 29 DEGs (*p* = 1.8 × 10^−6^, Figure S2), followed by “Focal adhesion” (28 DEGs, *p* = 6.1 × 10^−4^) and “Neuroactive ligand-receptor interaction” (28 DEGs, *p* = 7.4 × 10^−2^).

**Figure 7:**
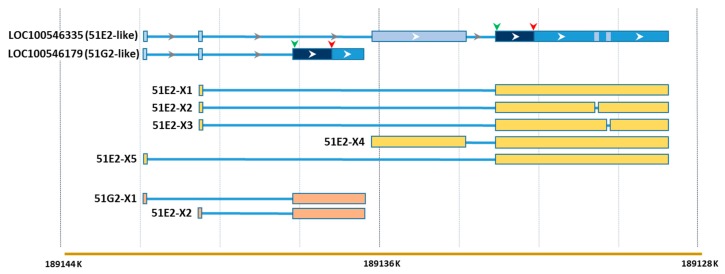
Alignment of NCBI predicted sequence variants to the predicted genes for two olfactory receptor loci.

Among the 1397 unique DEGs were two olfactory receptor genes, *LOC100546335* (*OR51E2*-like) and *LOC1005546179* (*OR51G2*-like). Both of these loci were up regulated in the EW birds compared to DT with AFB_1_-treatment (log_2_FC = 8.15 and 8.46, respectively). Expression of functional taste and olfactory receptors has been observed in human enteroendocrine cells [38,39] and a survey of RNAseq data from multiple human tissues identified expressed olfactory receptors with broad and tissue-exclusive expression [40]. An interesting aspect of *LOC100546335* and *LOC1005546179* is that based on read count, expression of both loci was roughly similar. These loci are adjacent in the turkey genome and are annotated as sharing two non-coding 5′ exons (Figure 7). A total of seven transcript variants for the two genes were predicted by NCBI’s automated computational analysis gene prediction method (Gnomon). Examination of RNAseq reads from 3 individuals in the present study (EW1, EW9 and NC11) found split RNAseq reads (intron spanning) that support each of the predicted variants with the exception of the variant 51E2- -X4. However, RNAseq reads did map to the non-coding upstream (5′) exon of variant 4 (Figure 7). Interestingly, split reads were also identified in each individual that indicated splicing events between the two small 5′ exons, not predicted in the NCBI models.

## 3. Discussion

Naturally-occurring dietary toxins such as AFB_1_ pose significant public health risk throughout the world but especially in locales characterized by high contamination levels of dietary staples such as corn. One of most significant is AFB_1_ which primarily targets the liver, the organ with the highest concentration of bioactivating CYPs. Extra-hepatic metabolism and bioactivation of this mycotoxin is a much-studied topic [41] but comparatively few studies have focused on the gastrointestinal tract, even though dietary exposure is the principal route for people and animals. Conversion of AFB_1_ to the AFBO epoxide has been implicated in the rat intestine [42] and even nasal mucosal cells [43]. Studies of cultured human intestinal epithelial cells (Caco-2) found AFB_1_ decreases trans-epithelial electrical resistance (TEER) [44]. Similarly, Romero et al. [45] reported that AFB_1_ treatment caused a reduction in TEER and mitochondrial viability and increased cell permeability. By contrast, the detoxified AFB_1_ metabolite AFM_1_ did not permanently compromise the integrity of Caco-2 cells grown on microporous filter supports [46]. In poultry, AFB_1_ is efficiently absorbed in the upper GI tract and thus exposure of the intestinal mucosa is greater than in other organs. While we have not quantified AFB_1_ bioactivation in the turkey gut, expression of the primary hepatic AFB_1_-activating *CYP1A5* was highly upregulated by AFB_1_ in the turkey cecum. Increased *CYP1A5* expression in AFB_1_-treated turkeys was also observed in the liver [17] and is a common observation in animals, as this and other CYPs are known to be induced by AFB_1_ and other foodborne and environmental toxicants [47]. Similarly, expression of GSTAs (particularly *GSTA4*), were up regulated by AFB_1_. In contrast, a prior study found expression of GSTAs in the liver were oppositely affected; *GSTA1*, *GSTA2* and *GSTA4* were down regulated after 2 weeks exposure to AFB_1_ and expression of *GSTA3* was significantly lower in EW birds compared to DT after AFB_1_ treatment [17].

The gastrointestinal epithelium provides an important physical barrier to foreign antigens and pathogens and disruptions thereof are increasingly associated with diseases [48]. Although few studies have specifically investigated the ability of aflatoxin to compromise intestinal permeability [19,49], the potential for mycotoxins to cause dysfunction of the intestinal barrier has come under increased study. Mycotoxins modulate the composition of gut microbiota, often eliminating beneficial bacteria, which leads to increased colonization by gut pathobionts and pathogens [50,51]. Exposure to AFB_1_ has been shown to induce changes in gut microbiota in rodents [52,53] and to modify barrier function in intestinal epithelial cells [49]. Probiotic gram-positive strains of *Lactobacillus*, *Propionibacterium* and *Bifidobacterium* have been proposed as feed additives to attenuate AFB_1_-induced toxicity in poultry due to their ability to bind AFB_1_, thereby reducing its bioavailability [54,55,56,57]. Gene expression in AFB_1_-treated birds is modulated by probiotics but the negative effects of AFB_1_ are not fully mitigated [15,16]. It is possible that in addition to binding AFB_1_, these probiotics exert positive effects by acting to decrease gut permeability and other protective functions [58].

Of interest in the present study is the potential of AFB_1_ to disrupt tight junction proteins allowing for increased translocation of substances from the lumen to the blood and lymphatic circulation [49]. Transmembrane tight junctions consist of claudins, occludin, tricellulin and a group of junction adhesion molecules that form the horizontal barrier at the apical lateral membrane [59]. Claudins are a family of transmembrane proteins that are essential components in the apical junctional complex of epithelia and endothelia cells [60], the expression of which in humans, is modulated by aflatoxins [45,61]. Romero et al. [45] found dose-dependent down regulation in *CLDN3* and occludin in human Caco-2 cells treated with AFB_1_ consistent with an observed decrease in gut barrier properties. Gao et al. [61] found decreased expression of TJ proteins (*CLDN3*, *CLDN4*, occludin and zonula occludens-1) and disrupted structures following exposure to aflatoxin M_1_ (4-hydroxylated metabolite of AFB_1_).

Dietary AFB_1_ treatment in the present study elicited transcriptional changes in several claudin transcripts including up regulation of *CLDN1* in both EW and DT, down regulation of *CLDN3* in DT, down regulation of *CLDN18* in EW and up regulation of *CLDN10* and *CLDN23* in EW birds. Transcriptional modifications of claudins may indicate a response to restore impaired TJ proteins and potentially compromised gut permeability. In vivo studies in poultry have produced inconsistent results. In broilers, AFB_1_ increased gut permeability as measured by the serum lactose/rhamnose ratio (dual sugar test), as well as increases in expression of *CLDN1*, multiple jejunal amino acid transporters and the translation initiation factor 4E [21]. A second study [20] found no evidence for increased gut permeability in broilers as measured by GI leakage of FITC-d following exposure to varying concentrations of AFB_1_. Annotation of avian claudin genes is based on similarities to mammalian orthologs and in many cases function has not been experimentally demonstrated. Results of the present study indicate that additional studies of the effect of AFB_1_ on gut permeability in turkey are needed.

Exposure to AFB_1_ has widespread adverse physiologic effects. In poultry, AFB_1_ adversely affects production characteristics causing poor performance, decreased growth rate, body weight, weight gain, egg production, reproductive performance and feed efficiency [62]. Humoral and cell-mediated immune functions in poultry are also impaired by AFB_1_ in keeping with its well-known immunotoxicity [3,5,6,16,41,63,64,65]. Altered humoral response to fowl cholera and Newcastle Disease (ND) virus has been described in chickens where correlation was observed between outbreaks of ND and AFB_1_-contaminated feeds (reviewed in Reference [65]). Effects on cell-mediated immunity are evident as decreased phagocytic activity in leukocytes [66,67,68,69]. Exposure to AFB_1_ in turkeys causes suppression of humoral and cellular immunity resulting in compromised immune response in hatchlings making them more susceptible to disease [6]. In this respect, AFB_1_ is a “force-multiplier” synergizing the adverse effects of other agents and pathogens detrimental to poultry health.

Compromised epithelial barrier is associated with increased paracellular permeability that may lead to overstimulation of the gut immune system and a non-specific systemic inflammatory response [48,70]. The cecal tonsil is the major lymphoid tissue in the avian cecum that provides important and unique immune functions. Detailed studies in poultry have demonstrated impairment of the normal function of the cecal tonsil caused by AFB_1_ through depletion of lymphocytes and lesions in the absorptive cells [71]. AFB_1_ significantly decreases intestinal IgA(+) cells and the expression of immunoglobulins in the intestinal mucosa [72]. Dietary AFB_1_ exposure decreases cell-mediated immunity while inducing the inflammatory response. Immune activation and inflammation result in mucosal recruitment of activated cells, modulated by cytokines. Cytokine-mediated dysfunction of tight junctions is important in gastrointestinal disease [48] as cytokines and other growth factors may act to alternatively decrease (e.g., IL-10) or increase (e.g., IL-6) gut permeability [58]. In the commercial DT birds, numerous pro-inflammatory cytokines, TGF-β and EGF were significantly down regulated by AFB_1_ treatment. In contrast, the interleukin 6 (*IL6R*) and interleukin 13, alpha 2 (*IL13RA2*) receptors and the interleukin 1 receptor accessory protein (*IL1RAP*) were significantly up regulated in both EW and DT birds. In humans, IL13RA2 functions to internalize the immunoregulatory cytokine IL-13. Dysregulation of IL6 impacts *CLDN2* expression (significantly up regulated by AFB_1_ in DT in this study) and can undermine the integrity of the intestinal barrier [73].

In response to the luminal environment, chemical receptors of intestinal epithelial and neuroendocrine cells modulate the function of these cells and ultimately systematic metabolism and homeostasis [38,74]. For example, ingestion of food results in signaling to the brain to regulate food intake and detection of bacterial metabolites may induce host defense responses. Part of this gut-brain axis is performed by enteroendocrine L-cells with specific nutrient-sensing receptors [30]. These include intestinal olfactory receptors that recognize ingested odor compounds and alter glucose homeostasis through induced secretion of gut-peptides [75]. In pigs, the olfactory receptor OR51E1 has been localized to enteroendocrine cells along the GI tract. Expression of the gene encoding this receptor was significantly altered following modulation of the intestinal microbiota, presumably in response to microbial metabolites [76]. Differential expression of OR genes in the turkey GIT may be caused by a direct action of AFB_1_ on the intestinal epithelial cells or secondarily through changes in the intestinal microbiota induced by AFB_1_.

Intensive breeding and genetic selection to produce the modern domesticated turkey has dramatically affected performance metrics. For example, growth rate to market age has essentially doubled in the past 40 years and feed efficiency of contemporary tom turkeys is approximately 50% better when compared to non-growth selected birds fed modern diets [77]. Under normal conditions, commercial birds typically reach 19 lbs. by 20 weeks of age, with a feed conversion ratio of approximately 2.5 [78]. Our results suggest that selection for production traits, such as increased nutrient conversion, may have contributed to the extreme sensitivity of DT to AFB_1_. In the same way, the relative resistance of WT, in addition to expression of AFB_1_-detoxifying GSTAs, may also involve extra-hepatic mechanisms such as a more refractory gastrointestinal tract, in addition to the presence of functional hepatic GST-mediated AFB_1_ detoxifying capability [12,13]. Possibly related to this, studies of production performance in chickens suggest that sensitivity to AFB_1_ has increased since the 1980s, concomitant with industry selection for increased nutrient conversion and demands for greater metabolism (reviewed in Yunus et al. [65]). Elucidation of extra-hepatic routes of pathogenesis provides a clearer picture of the complexity of species resistance and susceptibility to this potent mycotoxin that may also suggest analogous mechanisms in humans.

## 4. Materials and Methods 

This study used turkeys previously found to vary in AFB_1_-detoxifying GST activity. Animal husbandry and the AFB_1_ protocol were as described in Reed et al. [17]. Birds included AFB_1_-treated and control animals from the Eastern Wild (EW, *Meleagris gallopavo silvestris*) subspecies and domesticated Nicholas turkeys (DT). Male turkey poults were subjected to a short-term AFB_1_-treatment protocol in which the diet of challenge birds was supplemented beginning on day 15 of age with 320 ppb AFB_1_ and continued for 14 days. Previous studies with higher AFB_1_ dosing (1 ppm) caused an unacceptable mortality rate. Birds serving as experimental controls received a standard AFB_1_-free diet. At the end of the trial, birds were euthanized and a section of the cecum corresponding to the cecal tonsil was removed and placed in RNAlater (ThermoFisher Scientific, Waltham, MA, USA) for RNA isolation and RNAseq analysis. All procedures were approved by Utah State University’s Institutional Animal Use and Care Committee (Approval #2670, date of approve: 26 September 2016).

### 4.1. RNA Isolation and Sequencing

Total RNA was isolated from cecal tonsils by TRIzol extraction (ThermoFisher), treated with DNAse (Turbo DNA-freeTM Kit, ThermoFisher) and stored at −80° C. Library preparation and sequencing was performed at the University of Minnesota Genomics Center. Briefly, concentration and quality of RNA was assessed on a 2100 Bioanalyzer (Agilent Technologies) and RNA Integrity Numbers (RIN) averaged 6.7. Replicate samples (*n* = 4) from each treatment group were examined. Indexed libraries (*n* = 16) were constructed, multiplexed, pooled and sequenced (101-bp paired-end reads) on the HiSeq 2000 using v3 chemistry (Illumina, Inc., San Diego, CA, USA). Sequence reads were groomed, assessed for quality and mapped to turkey genome (UMD 5.0, NCBI Annotation 101) as described in Reed et al. [17].

### 4.2. Quantitative Real-Time PCR

Quantitative real-time PCR (qRT-PCR) was performed on both domesticated and wild turkeys. Samples included the Eastern Wild (EW; *M. g. silvestris*) and domesticated Nicholas turkey (DT) birds, plus domesticated Broad Breasted White (BB) and birds of the Rio Grande subspecies of wild turkey (RGW; *M. g. intermedia*) from a parallel AFB_1_-challenge experiment. Of the 6 samples from the DT and EW groups used for qRT-PCR, four were in common with the RNAseq study. Synthesis of cDNA was performed on DNase-treated mRNA using Invitrogen Super Script IV First-strand synthesis kit (Invitrogen, Carlsbad, CA, USA). The iTaq Universal SYBR Green Supermix (BioRad, Hercules, CA, SA) was used for quantitative analysis of gene-specific amplicons with the CFX96 touch real time detection system (BioRad, Hercules, CA, USA). Primers were designed using the turkey genome sequence (UMD5.0) and Primer3 software [79]. Primer sets were designed so the amplicon spanned an exon/exon junction and at least one intron. Several normalizing genes were tested for uniformity and the most stable reference gene (hypoxanthine guanine phosphoribosyl transferase, *HPRT*) was determined with RefFinder [80]. Target gene reactions were conducted in triplicate and *HPRT* normalization reactions, no template and gDNA controls were run in duplicate. Disassociation curves were used to confirm single product amplification and to preclude the possibility of dimer amplification.

### 4.3. Statistical Analysis

For expression analysis of RNAseq data, read counts were by-total normalized and expressed as reads per 11.9M (CLC Genomics Workbench v. 8.0.2, CLC Bio, Aarhus, Denmark). Principal component analysis (PCA) and hierarchical clustering of samples based on Euclidean distance was performed (with single linkage) in CLCGWB using by-total normalization. Empirical analysis of differential gene expression (EdgeR) and ANOVA were performed in CLCGWB on mapped read counts with TMM (Trimmed Mean of M-values) normalization (Bonferroni and FDR corrected). Pair-wise comparisons between treatment groups were made following the standard workflow Wald test. Significant differentially expressed (DE) genes were used to investigate affected gene pathways with Ingenuity Pathway Analysis (IPA) (Ingenuity Systems, Redwood City, CA, USA). Gene Ontology (GO) and functional classification was performed in DAVID (v6.8, [81]) and overrepresentation tests for gene enrichment were performed with PANTHER (GO Consortium release 20150430) [82]. For analysis of qRT-PCR data, expression was normalized first to HPRT, then interpreted using the Double Delta Ct Analysis (ΔΔCt, [83]) and a comparative Ct approach. Expression analysis was performed using the standard ΔΔCt workflow within the CFX Maestro software package (Biorad, Hercules, CA, USA).

## Figures and Tables

**Table 1:** Summary of genes with significant differential expression (DE) in pair-wise comparisons of treatment groups.

Comparison	Groups	Expressed Genes	Shared Genes	Unique Genes (Each Group)	FDR *p*-Value < 0.05	|log_2_FC| > 1.0	|log_2_FC| > 2.0	Up/Down Regulated
AFB_1_	EW (AFB vs. CNTL)	18744	17833	402/509	703	703	687	674/13
	DT (AFB vs. CNTL)	18654	16277	304/2073	11237	7568	4515	1563/2952
Line	CNTL (EW vs. DT)	18736	17956	386/394	679	348	67	37/30
	AFB (EW vs. DT)	18447	16369	1866/212	1666	1666	1410	1308/102

For each comparison, the treatment groups, total number of expressed, shared and unique genes, genes with significant FDR *p*-value and the numbers of significant DE genes that also had |log_2_ fold change| >1.0 and >2.0 are given. For the DE genes with |log_2_ fold change| > 2.0 the number of genes up and down regulated are given. Genes were considered expressed in a treatment group if by-total normalized read count ≥ 3.0 in any individual within the group.

## Supplementary Materials

The following are available online at http://www.mdpi.com/2072-6651/11/1/55/s1. Figure S1: Hierarchical clustering of samples based on Euclidean distance reiterated relationships shown by PCA. Figure S2: Kegg calcium-signaling pathway. Table S1: Summary of RNAseq data for turkey cecal tonsil transcriptomes. Table S2: Mean quality-trimmed RNAseq read counts for turkey cecal tonsil from two turkey types (Wild and Domesticated). Table S3: Summary of pairwise differential gene expression analysis of cecal tonsil transcriptomes. Table S4: Fifty genes showing the greatest differential expression in each pairwise comparison of treatment groups. Table S5: Functional annotation gene clusters identified in DAVID among the 655 DEGs shared between EW and DT birds in AFB_1_ versus CNTL comparisons. Table S6: Significant differentially expressed genes (FDR *p*-values < 0.05 and |log_2_FC| > 2.0) identified in comparison of Eastern Wild versus domesticated turkeys in the CNTL groups. Table S7: Genes showing differential expression that were unique in the comparison of AFB_1_-treated Eastern wild turkeys versus domesticated turkeys.

## Abbreviations

AFB	aflatoxin B_1_
AFBO	exo-AFB1-8,9-epoxide
BB	Broad Breasted White
Ct	threshold cycle
CYP	cytochrome P450
DE	differentially expressed
DEG	differentially expressed gene
DT	domesticated turkey
EW	Eastern wild turkey (*Meleagris gallopavo silvestris*)
FC	fold change
FDR	false discovery rate
GO	gene ontology
GST	glutathione S-transferase
IPA	Ingenuity Pathway Analysis
ncRNA	non-coding RNA
PCA	principal component analysis
qRT-PCR	quantitative real-time polymerase chain reaction
RGW	Rio Grande wild turkey (*Meleagris gallopavo intermedia*)

## References

[1] CAST (2003). Mycotoxins: Risks in Plant, Animal and Human Systems.

[2] Blount W.P. (1961). Turkey “X” disease. Turkeys.

[3] Rawal S., Kim J.E., Coulombe R. (2010). Aflatoxin B_1_ in poultry: Toxicology, metabolism and prevention. Res. Vet. Sci..

[4] Monson M.S., Coulombe R.A., Reed K.M. (2015). Aflatoxicosis: Lessons from toxicity and responses to aflatoxin B_1_ in poultry. Agriculture.

[5] Qureshi M.A., Brake J., Hamilton P.B., Hagler W.M., Nesheim S. (1998). Dietary exposure of broiler breeders to aflatoxin results in immune dysfunction in progeny chicks. Poult. Sci..

[6] Qureshi M.A., Heggen C.L., Hussain I. (2000). Avian macrophage: Effector functions in health and disease. Dev. Comp. Immunol..

[7] Williams J.G., Deschl U., Williams G.M. (2011). DNA damage in fetal liver cells of turkey and chicken eggs dosed with aflatoxin B_1_. Arch. Toxicol..

[8] Quist C.F., Bounous D.I., Kilburn J.V., Nettles V.F., Wyatt R.D. (2000). The effect of dietary aflatoxin on wild turkey poults. J. Wildl. Dis..

[9] Rawal S., Coulombe R.A. (2011). Metabolism of aflatoxin B_1_ in turkey liver microsomes: The relative roles of cytochromes P450 1A5 and 3A37. Toxicol. Appl. Pharmacol..

[10] Bunderson B.R., Kim J.E., Croasdell A., Mendoza K.M., Reed K.M., Coulombe R.A. (2013). Heterologous expression and functional characterization of avian mu-class glutathione S-transferases. Comp. Biochem. Physiol. C Toxicol. Pharmacol..

[11] Kim J.E., Bunderson B.R., Croasdell A., Coulombe R.A. (2011). Functional characterization of alpha-class glutathione *S*-transferases from the turkey (*Meleagris gallopavo*). Toxicol. Sci..

[12] Kim J.E., Bunderson B.R., Croasdell A., Reed K.M., Coulombe R.A. (2013). Alpha-class glutathione *S*-transferases in wild turkeys (*Meleagris gallopavo*): Characterization and role in resistance to the carcinogenic mycotoxin aflatoxin B_1_. PLoS ONE.

[13] Klein P.J., Buckner R., Kelly J., Coulombe R.A. (2000). Biochemical basis for the extreme sensitivity of turkeys to aflatoxin B_1_. Toxicol. Appl. Pharmacol..

[14] Klein P.J., Van Vleet T.R., Hall J.O., Coulombe R.A. (2002). Dietary butylated hydroxytoluene protects against aflatoxicosis in turkeys. Toxicol. Appl. Pharmacol..

[15] Monson M.S., Settlage R.E., McMahon K.W., Mendoza K.M., Rawal S., El-Nemazi H.S., Coulombe R.A., Reed K.M. (2014). Response of the hepatic transcriptome to aflatoxin B_1_ in domestic turkey (*Meleagris gallopavo*). PLoS ONE.

[16] Monson M.S., Settlage R.E., Mendoza K.M., Rawal S., El-Nezami H.S., Coulombe R.A., Reed K.M. (2015). Modulation of the spleen transcriptome in domestic turkey (*Meleagris gallopavo*) in response to aflatoxin B_1_ and probiotics. Immunogenetics.

[17] Reed K.M., Mendoza K.M., Abrahante J.E., Coulombe R.A. (2018). Comparative response of the hepatic transcriptomes of domesticated and wild turkey to aflatoxin B_1_. Toxins.

[18] Monson M.S., Cardona C.C., Coulombe R.A., Reed K.M. (2016). Hepatic transcriptome responses of domesticated and wild turkey embryos to aflatoxin B_1_. Toxins.

[19] Grenier B., Applegate T.J. (2013). Modulation of intestinal functions following mycotoxin ingestion: Meta-analysis of published experiments in animals. Toxins.

[20] Galarza-Seeber R., Latorre J.D., Bielke L.R., Kuttappan V.A., Wolfenden A.D., Hernandez-Velasco X., Merino-Guzman R., Vicente J.L., Donoghue A., Cross D. (2016). Leaky gut and mycotoxins: Aflatoxin B_1_ does not increase gut permeability in broiler chickens. Front. Vet. Sci..

[21] Chen X., Naehrer K., Applegate T.J. (2016). Interactive effects of dietary protein concentration and aflatoxin B_1_ on performance, nutrient digestibility and gut health in broiler chicks. Poult. Sci..

[22] Stanley D., Denman S.E., Hughes R.J., Geier M.S., Crowley T.M., Chen H., Haring V.R., Moore R.J. (2012). Intestinal microbiota associated with differential feed conversion efficiency in chickens. Appl. Microbiol. Biotechnol..

[23] Sasaki H., Matsui C., Furuse K., Mimori-Kiyosue Y., Furuse M., Tsukita S. (2003). Dynamic behavior of paired claudin strands within apposing plasma membranes. Proc. Natl. Acad. Sci. USA.

[24] Grishin A.V., Sverdlov V.E., Kostina M.B., Modyanov N.N. (1994). Cloning and characterization of the entire cDNA encoded by ATP1AL1--a member of the human Na,K/H,K-ATPase gene family. FEBS Lett..

[25] Hinson E.R., Cresswell P. (2009). The N-terminal amphipathic alpha-helix of viperin mediates localization to the cytosolic face of the endoplasmic reticulum and inhibits protein secretion. J. Biol. Chem..

[26] Wang J., Yu L., Schmidt R.E., Su C., Huang X., Gould K., Cao G. (2005). Characterization of HSCD5, a novel human stearoyl-CoA desaturase unique to primates. Biochem. Biophys. Res. Commun..

[27] Grahn T.H., Zhang Y., Lee M.J., Sommer A.G., Mostoslavsky G., Fried S.K., Greenberg A.S., Puri V. (2013). FSP27 and PLIN1 interaction promotes the formation of large lipid droplets in human adipocytes. Biochem. Biophys. Res. Commun..

[28] Feild J.A., Zhang L., Brun K.A., Brooks D.P., Edwards R.M. (1999). Cloning and functional characterization of a sodium-dependent phosphate transporter expressed in human lung and small intestine. Biochem. Biophys. Res. Commun..

[29] Yan M., Brady J.R., Chan B., Lee W.P., Hsu B., Harless S., Cancro M., Grewal I.S., Dixit V.M. (2001). Identification of a novel receptor for B lymphocyte stimulator that is mutated in a mouse strain with severe B cell deficiency. Curr. Biol..

[30] Spreckley E., Murphy K.G. (2015). The L-cell in nutritional sensing and the regulation of appetite. Front. Nutr..

[31] Karniski L.P., Aronson P.S. (1985). Chloride/formate exchange with formic acid recycling: A mechanism of active chloride transport across epithelial membranes. Proc. Natl. Acad. Sci. USA.

[32] Racioppi L., Means A.R. (2008). Calcium/calmodulin-dependent kinase IV in immune and inflammatory responses: Novel routes for an ancient traveler. Trends Immunol..

[33] Zhang Y., Burke C.W., Ryman K.D., Klimstra W.B. (2007). Identification and characterization of interferon-induced proteins that inhibit alphavirus replication. J. Virol..

[34] Mulero J.J., Boyle B.J., Bradley S., Bright J.M., Nelken S.T., Ho T.T., Mize N.K., Childs J.D., Ballinger D.G., Ford J.E. (2002). Three new human members of the lipid transfer/lipopolysaccharide binding protein family (LT/LBP). Immunogenetics.

[35] Sperandio V., Torres A.G., Jarvis B., Nataro J.P., Kaper J.B. (2003). Bacteria-host communication: The language of hormones. Proc. Natl. Acad. Sci. USA.

[36] Prabhu A.V., Luu W., Sharpe L.J., Brown A.J. (2017). Phosphorylation regulates activity of 7-dehydrocholesterol reductase (DHCR7), a terminal enzyme of cholesterol synthesis. J. Steroid Biochem. Mol. Biol..

[37] Roach P.J., Skurat A.V. (1997). Self-glucosylating initiator proteins and their role in glycogen biosynthesis. Prog. Nucl. Acid Res. Mol. Biol..

[38] Braun T., Voland P., Kunz L., Prinz C., Gratzl M. (2007). Enterochromaffin cells of the human gut: Sensors for spices and odorants. Gastroenterology.

[39] Sternini C., Anselmi L., Rozengurt E. (2008). Enteroendocrine cells: A site of ‘taste’ in gastrointestinal chemosensing. Curr. Opin. Endocrinol. Diabetes Obes..

[40] Flegel C., Manteniotis S., Osthold S., Hatt H., Gisselmann G. (2013). Expression profile of ectopic olfactory receptors determined by deep sequencing. PLoS ONE.

[41] Coulombe R.A. (1993). Biological action of mycotoxins. J. Dairy Sci..

[42] Kumagai S. (1989). Intestinal absorption and excretion of aflatoxin in rats. Toxicol. Appl. Pharmacol..

[43] Larsson P., Tjälve H. (2000). Intranasal instillation of aflatoxin B_1_ in rats: Bioactivation in the nasal mucosa and neuronal transport to the olfactory bulb. Toxicol. Sci..

[44] Gratz S., Wu Q.K., El-Nezami H., Juvonen R.O., Mykkänen H., Turner P.C. (2007). *Lactobacillus rhamnosus* strain GG reduces aflatoxin B_1_ transport, metabolism and toxicity in Caco-2 cells. Appl. Environ. Microbiol..

[45] Romero A., Ares I., Ramos E., Castellano V., Martínez M., Martínez-Larrañaga M.R., Anadón A., Martínez M.A. (2016). Mycotoxins modify the barrier function of Caco-2 cells through differential gene expression of specific claudin isoforms: Protective effect of illite mineral clay. Toxicology.

[46] Caloni F., Cortinovis C., Pizzo F., De Angelis I. (2012). Transport of aflatoxin M_1_ in human intestinal caco-2/TC7 cells. Front. Pharmacol..

[47] Chang S.Y., Voellinger J.L., Van Ness K.P., Chapron B., Shaffer R.M., Neumann T., White C.C., Kavanagh T.J., Kelly E.J., Eaton D.L. (2017). Characterization of rat or human hepatocytes cultured in microphysiological systems (MPS) to identify hepatotoxicity. Toxicol. In Vitro.

[48] Camilleri M., Madsen K., Spiller R., Van Meerveld B.G., Verne G.N. (2012). Intestinal barrier function in health and gastrointestinal disease. Neurogastroenterol. Motil..

[49] Akbari P., Braber S., Varasteh S., Alizadeh A., Garssen J., Fink-Gremmels J. (2017). The intestinal barrier as an emerging target in the toxicological assessment of mycotoxins. Arch. Toxicol..

[50] Liew W.P., Mohd-Redzwan S. (2018). Mycotoxin: Its impact on gut health and microbiota. Front. Cell Infect. Microbiol..

[51] Robert H., Payros D., Pinton P., Théodorou V., Mercier-Bonin M., Oswald I.P. (2017). Impact of mycotoxins on the intestine: Are mucus and microbiota new targets?. J. Toxicol. Environ. Health B Crit. Rev..

[52] Ishikawa A.T., Weese J.S., Bracarense A.P.F.R.L., Alfieri A.A., Oliveira G.G., Kawamura O., Hirooka E.Y., Itano E.N., Costa M.C. (2017). Single aflatoxin B_1_ exposure induces changes in gut microbiota community in C57Bl/6 mice. World Mycotoxin J..

[53] Wang J., Tang L., Glenn T.C., Wang J.S. (2016). Aflatoxin B_1_ induced compositional changes in gut microbial communities of male F344 rats. Toxicol. Sci..

[54] El-Nezami H., Kankaanpaa P., Salminen S., Ahokas J. (1998). Ability of dairy strains of lactic acid bacteria to bind a common food carcinogen, aflatoxin B_1_. Food Chem. Toxicol..

[55] Gratz S., Mykkänen H., El-Nezami H. (2005). Aflatoxin B_1_ binding by a mixture of *Lactobacillus* and *Propionibacterium*: In vitro versus ex vivo. J. Food Prot..

[56] Kankaanpää P., Tuomola E., El-Nezami H., Ahokas J., Salminen S.J. (2000). Binding of aflatoxin B_1_ alters the adhesion properties of *Lactobacillus rhamnosus* strain GG in a Caco-2 model. J. Food Prot..

[57] Oatley J.T., Rarick M.D., Ji G.E., Linz J.E. (2000). Binding of aflatoxin B_1_ to *Bifidobacteria* in vitro. J. Food Prot..

[58] Suzuki T. (2013). Regulation of intestinal epithelial permeability by tight junctions. Cell. Mol. Life Sci..

[59] Tsukita S., Furuse M., Itoh M. (2001). Multifunctional strands in tight junctions. Nat. Rev. Mol. Cell Biol..

[60] Tsukita S., Furuse M. (2000). The structure and function of claudins, cell adhesion molecules at tight junctions. Ann. N. Y. Acad Sci..

[61] Gao Y., Songli L., Wang J., Luo C., Zhao S., Zheng N. (2018). Modulation of intestinal epithelial permeability in differentiated Caco-2 cells exposed to aflatoxin M_1_ and ochratoxin A individually or collectively. Toxins.

[62] Pandey I., Chauhan S.S. (2007). Studies on production performance and toxin residues in tissues and eggs of layer chickens fed on diets with various concentrations of aflatoxin AFB_1_. Br. Poult. Sci..

[63] Giambrone J.J., Ewert D.L., Wyatt R.D., Eidson C.S. (1978). Effect of aflatoxin on the humoral and cell-mediated immune systems of the chicken. Am. J. Vet. Res..

[64] Hoerr F.J. (2010). Clinical aspects of immunosuppression in poultry. Avian Dis..

[65] Yunus A.W., Razzazi-Fazeli E., Bohm J. (2011). Aflatoxin B_1_ in affecting broiler’s performance, immunity and gastrointestinal tract: A review of history and contemporary issues. Toxins.

[66] Chang C.F., Hamilton P.B. (1979). Impaired phagocytosis by heterophils from chickens during aflatoxicosis. Toxicol. Appl. Pharmacol..

[67] Chang C.F., Hamilton P.B. (1979). Impairment of phagocytosis in chicken monocytes during aflatoxicosis. Poult. Sci..

[68] Ghosh R.C., Chauhan H.V., Jha G.J. (1991). Suppression of cell-mediated immunity by purified aflatoxin B_1_ in broiler chicks. Vet. Immunol. Immunopathol..

[69] Neldon-Ortiz D.L., Qureshi M.A. (1992). Effects of AFB_1_ embryonic exposure on chicken mononuclear phagocytic cell functions. Dev. Comp. Immunol..

[70] König J., Wells J., Cani P.D., García-Ródenas C.L., MacDonald T., Mercenier A., Whyte J., Troost F., Brummer R.J. (2016). Human intestinal barrier function in health and disease. Clin. Transl. Gastroenterol..

[71] Liu C., Zuo Z., Zhu P., Zheng Z., Peng X., Cui H., Zhou Y., Ouyang P., Geng Y., Deng J. (2017). Sodium selenite prevents suppression of mucosal humoral response by AFB_1_ in broiler’s cecal tonsil. Oncotarget.

[72] Jiang M., Fang J., Peng X., Cui X., Yu Z. (2015). Effect of aflatoxin B_1_ on IgA+ cell number and immunoglobulin mRNA expression in the intestine of broilers. Immunopharmacol. Immunotoxicol..

[73] Suzuki T., Yoshinaga N., Tanabe S. (2011). Interleukin-6 (IL-6) regulates claudin-2 expression and tight junction permeability in intestinal epithelium. J. Biol. Chem..

[74] Kaji I., Karaki S., Kuwahara A. (2014). Taste sensing in the colon. Curr. Pharm. Des..

[75] Kim K.S., Lee I.S., Kim K.H., Park J., Kim Y., Choi J.H., Choi J.S., Jang H.J. (2017). Activation of intestinal olfactory receptor stimulates glucagon-like peptide-1 secretion in enteroendocrine cells and attenuates hyperglycemia in type 2 diabetic mice. Sci. Rep..

[76] Priori D., Colombo M., Clavenzani P., Jansman A.J., Lallès J.P., Trevisi P., Bosi P. (2015). The olfactory receptor OR51E1 is present along the gastrointestinal tract of pigs, co-localizes with enteroendocrine cells and is modulated by intestinal microbiota. PLoS ONE.

[77] Havenstein G.B., Ferket P.R., Grimes J.L., Qureshi M.A., Nestor K.E. (2007). Comparison of the performance of 1966- versus 2003-type turkeys when fed representative 1966 and 2003 turkey diets: Growth rate, livability and feed conversion. Poult. Sci..

[78] Swalander M. (2015). Aspects of feed efficiency and feeding behaviour in turkeys. Aviagen Turk. Manag..

[79] Untergasser A., Cutcutache I., Koressaar T., Ye J., Faircloth B.C., Remm M., Rozen S.G. (2012). Primer3-new capabilities and interfaces. Nucleic Acids Res..

[80] Xie F., Xiao P., Chen D., Xu L., Zhang B. (2012). miRDeepFinder: A miRNA analysis tool for deep sequencing of plant small RNAs. Plant Mol. Biol..

[81] Huang D.W., Sherman B.T., Lempicki R.A. (2009). Systematic and integrative analysis of large gene lists using DAVID bioinformatics resources. Nat. Protoc..

[82] Mi H., Muruganujan A., Thomas P.D. (2013). PANTHER in 2013: Modeling the evolution of gene function and other gene attributes, in the context of phylogenetic trees. Nucleic Acids Res..

[83] Schmittgen T.D., Livak K.J. (2008). Analyzing real-time PCR data by the comparative C(T) method. Nat. Protoc..

