# Supplementary material for: Differential Transcriptome Responses to Aflatoxin B1 in the Cecal Tonsil of Susceptible and Resistant Turkeys
Source: Toxins (Basel). 2019 Jan 18;11(1):55. doi: 10.3390/toxins11010055 (PMC6357151; doi:10.3390/toxins11010055)
Supplement: Supplementary file 1 [file toxins-11-00055-s001.zip › toxins-413334-supplementary/toxins-413314-supple-final/toxins-413314-supple-final.pdf]

Supplementary Materials: Differential Transcriptome Responses to Aflatoxin B_1 in the Cecal Tonsil of Susceptible and Resistant Turkeys

Kent M. Reed, Kristelle M. Mendoza and Roger A. Coulombe, Jr.

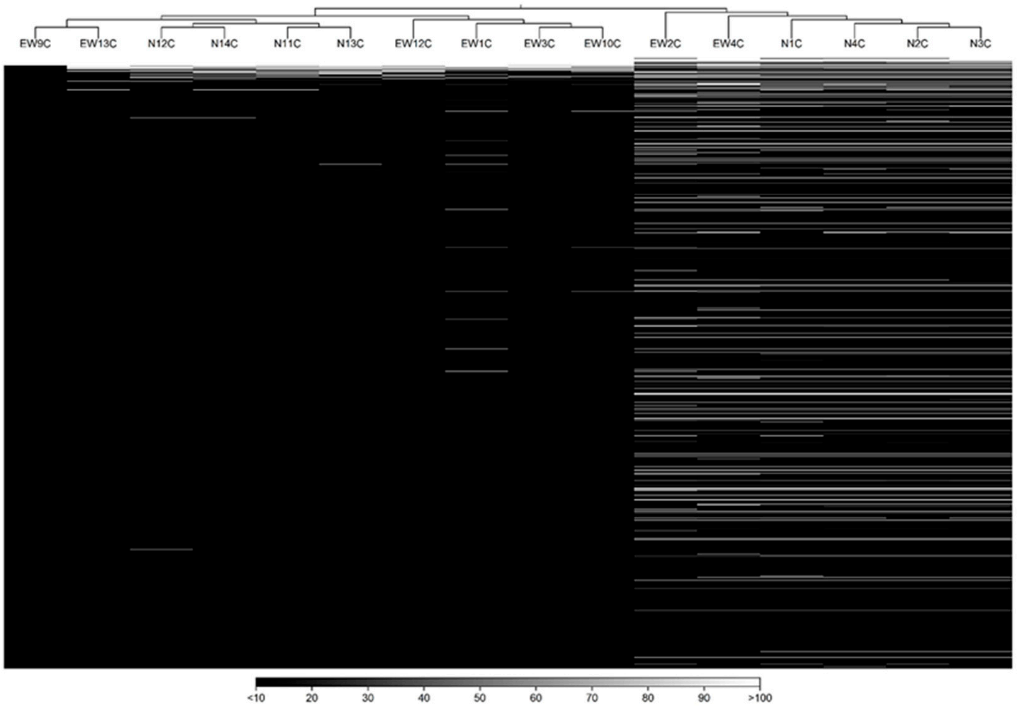

Figure S1. Hierarchical clustering of samples based on Euclidean distance reiterated relationships shown by PCA.

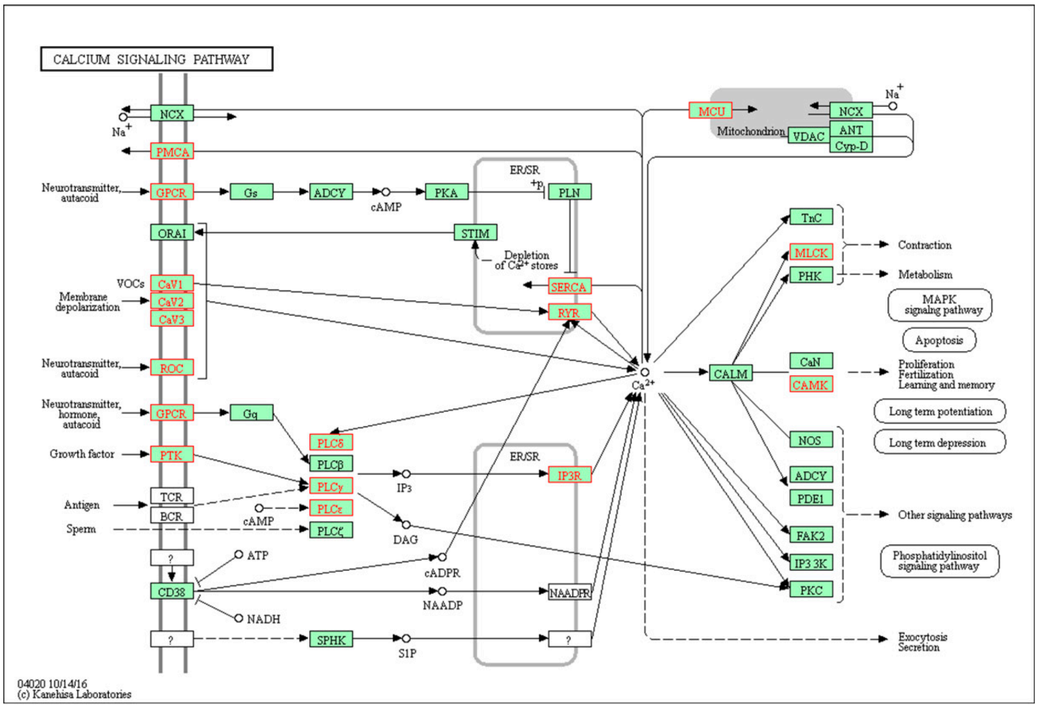

Figure S2. Kegg calcium-signaling pathway.
